# Perceived urban green spaces and youth mental health in the post-COVID-19 era

**DOI:** 10.3389/fpubh.2024.1265682

**Published:** 2024-02-07

**Authors:** Mahsa Mollaesmaeili, Pantea Hakimian, Azadeh Lak

**Affiliations:** Faculty of Architecture and Urban Planning, Shahid Beheshti University, Tehran, Iran

**Keywords:** youth, UGSs, COVID-19, mental health, Isfahan

## Abstract

**Introduction:**

The urban green space (UGS) is one of the most significant urban spaces with unique visual and social features, including pleasant air, low noise, and vitality, making it a recreational place for citizens, especially the youth. According to previous studies, perceived green space and the interaction with it is associated with mental health and lower symptoms of anxiety and depression. Although the presence of urban and blue-green spaces in Isfahan has a long history, the UGSs have been out of reach, causing a significant impact on youth mental health due to the spread of COVID-19 and the forcing of the Iranian government to severe and long-term lockdown. This study investigates the relationship between the long-term isolation of youth and being away from UGSs on their mental health in Isfahan city.

**Methods:**

In September 2022, the youth (*n* = 273) in 12 neighborhoods with similar socio-economic status were asked to answer the online questionnaire. To investigate the correlation between perceived UGS and the mental health of the youth, Structural Equation Modeling (SEM) is done.

**Results:**

The results show that the perceived UGSs negatively relate to the youth’s fear of the reoccurrence of COVID-19 infection, anxiety, and depression. Moreover, the model shows that perceived UGS has an inverse correlation with anxiety (*β* = −0.24, *p* = 0.00), and no meaningful correlation exists with depression.

**Discussion:**

These results point to a practical solution for designing UDGs in residential areas for youth according to their benefits for mental health during the epidemic era.

## 1 Introduction

The spread of COVID-19 unexpectedly changed the routines, habits, and types of youth activities (3), causing highly noticeable consideration in densely populated cities and especially metropolises. During this period, quarantine and social distancing, two methods of dealing with the pandemic, led to social isolation, a high risk of depression, inactivity, and limited access to essential services (4).

However, many studies have found that exposure to Urban Green Spaces (UGSs) is associated with mental relaxation and improves mental health in youth (5–10). Kaplans” *Attention Restoration Theory* (11) and Ulrich” ‘s *Psycho-evolutionary Model* (12) were pioneering research investigating nature” ‘s effects on humans” physical and mental health. Many studies have recently investigated how UGSs significantly affect youth mental health (13–15).

Before the spread of COVID-19, UGSs, such as parks, have provided residents with a very high quality of life (3, 16), improving mental health and reducing depression and anxiety (17–19). Unfortunately, during the COVID-19 outbreak, social distancing prevented people from visiting UGSs and engaging in recreational activities (20). The policy of “stay at home” and confinement led to the isolation of millions of people all over the world (21–23), particularly the youth (3, 24). (20).

In the last few decades, the expansion of urbanization by reducing the connection between people and nature has unintentionally led to an increase in the risk of ‘citizens’ mental disorders (25–27). Moreover, most city residents are youth, and more than 80% of mental disorders occur before age 26 (13, 28). In 2019, one in every eight people were living with a mental disorder, with anxiety and depressive disorders the most common (29). In 2020, initial estimates showed a 26 and 28% increase, respectively, for anxiety and major depressive disorders in just 1 year (29). Nevertheless, people became more prone to show different degrees of stress and depression during COVID-19 (30–32) and the paucity of people’s interaction with UGSs based on the fear of COVID-19 infection (33–35) exacerbated their mental status.

During COVID-19, citizens had to use private open green spaces inside their residences (23, 30) due to the prohibition of using parks and playgrounds (36). In the post-COVID-19 era, the youth in urban areas have found the opportunity to interact with each other in UGSs and perform their daily activities without restrictions. However, few studies in the literature investigated the role of UGSs in the youth’s mental health after COVID-19, particularly in arid regions where the scares UGS is a challenge.

Therefore, this study aims to examine the relationship between UGSs and anxiety and depression among the youth in Isfahan, one of the megacities in Iran with the prominent feature of blue space, Zayandeh Rood, in the post-COVID-19 era. Accordingly, after developing the conceptual framework, we tried not only to identify the relationship between perceived UGSs in neighborhoods and the mental health of Isfahani youth but also to understand the relationships between the perceived aesthetic of UGSs, perceived pollution (air and noise), and fear of COVID-19 infection, as factors mediating this relationship with the structural equation model.

## 2 Theoretical framework

The United Nations defines youth as adolescents between 15 and 24 years old (37). According to Marketta Kyttä, the priority of children and youth-friendly cities is their freedom and independence (38). However, to reduce the risk of infection during COVID-19, the youth, like others, have been forced to stay at home, being highly susceptible to anxiety and depression at both immediate and long-term levels (13).

The main features of UGSs are their social environment and aesthetic aspects, which encourage people to visit them. Many studies point out that exposure to UGSs positively relates to the youths’ psychological well-being (11, 39). In addition, perceived aesthetics in UGSs plays an essential role in attending, performing activities, and socializing with others (40–42). Driskell (43) believes that the presence of green areas, contact with nature, clean air, and the absence of garbage are some physical characteristics of a good place for children and youth. In addition, physical access to UGSs is directly associated with the youth’s mental health (44, 45). Many local authorities have developed policy plans, including environmental education classes and adding outdoor games to the curriculum to improve the mental health of their children and youth (46).

While air pollution and noise are two influential factors in the youth’s mental health (15, 47, 48), UGSs can potentially reduce the effect of these harmful factors (17), (49–51). Although perceived air and noise pollution are most closely related to the pollution calculated by meteorological stations, it is much more intense than the actual level of perception, demonstrating the effect of the personality, social, or physical dimensions of the respondent’s residence (52). However, perceived pollution is more related to the youth’s behavior than measured pollution (26).

Although visiting UGSs could be considered an effective way to improve mental health (44, 45), during COVID-19, using UGSs has been associated with problems. In this period, social networks and communication channels gave people quick access to the news, provoking fear of this disease, hopelessness, and uncertainty (14). During the pandemic, people worldwide experienced many fears related to COVID-19, including the fear of being in urban public spaces. Despite considering all the positive factors that may encourage the youth to visit UGSs, fear of COVID-19 infection and the possibility of death were some of the major factors that prevent them from being in UGSs (35).

While perceived aesthetics are one of the positive factors in increasing the presence of the youth and encouraging them to use UGSs, two factors, perceived pollution and fear of COVID-19 infection, are some inhibiting factors in this research. According to the role of UGSs in reducing air and noise pollution (53–55) as well as in improving youth mental health (56, 57), we aimed to examine the link between the perceived UGSs and the youth” mental health during the COVID-19 pandemic.

**Figure 1:**
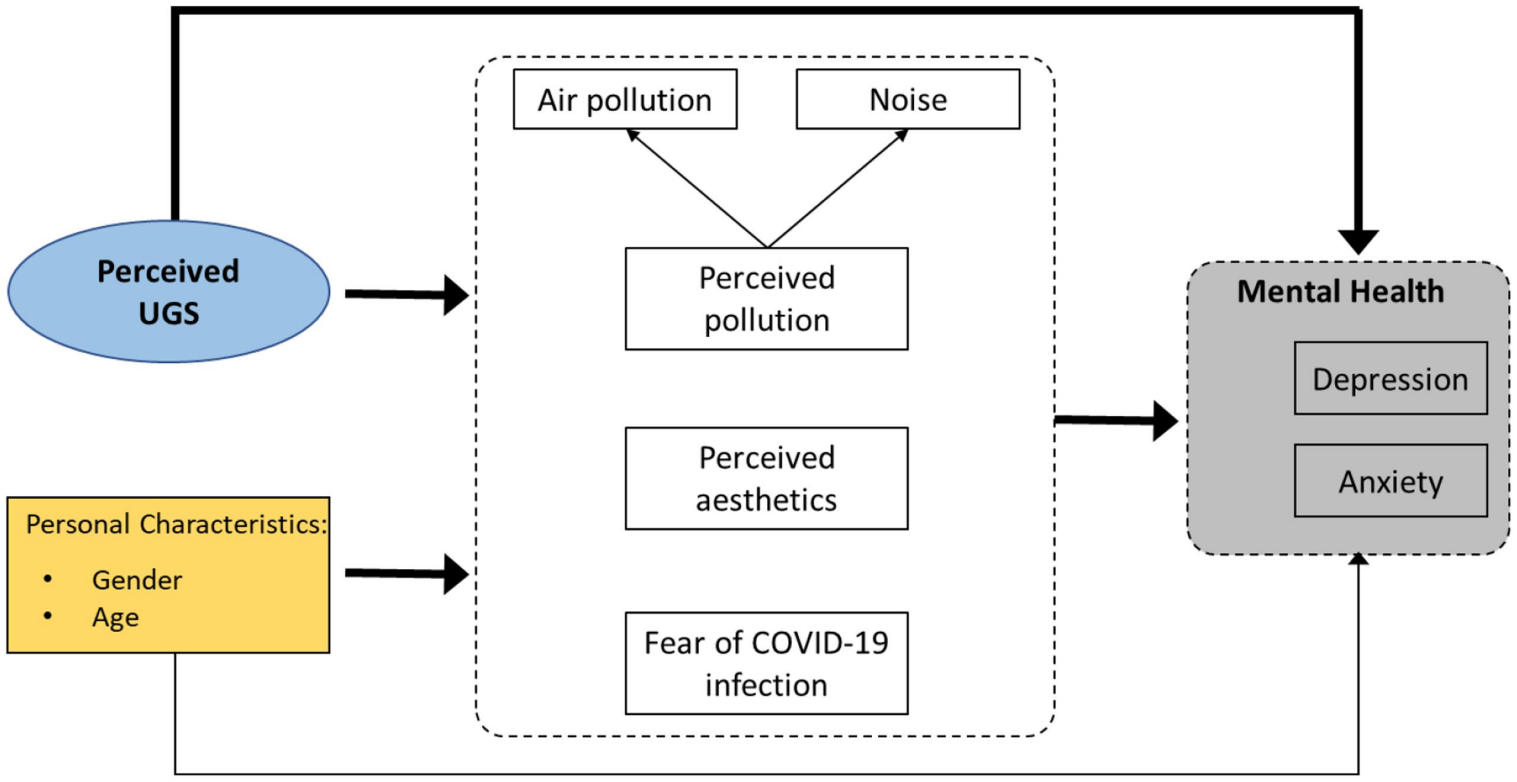
Conceptual framework. The pathways between the core variables (perceived UGS, perceived pollution, perceived aesthetics, fear of COVID-19 infection, depression, and anxiety) and control variables (age and gender) are demonstrated.

Based on the conceptual framework (Figure 1), our assumptions are as follows: (H1) To what extent are UGSs associated with youth mental health, specifically depression and anxiety, in the post-COVID-19 era? (H2) How do the perceived UGSs relate to the youth’s mental health through the three dependent variables of perceived aesthetics, perceived pollution (air and noise), and fear of COVID-19 infection?

## 3 Materials and methods

### 3.1 Study design and participants

The cross-sectional research was conducted in September 2022. According to the Worldomenter website, when this research was conducted, it had been about a year since the worst peak of COVID-19 in September, and the risk of infecting and dying from COVID-19 has recently decreased worldwide (58). According to the Ministry of Health of Iran, we started our study after finishing the 7th peak in August 2022; people have returned to their regular routines.

**Figure 2:**
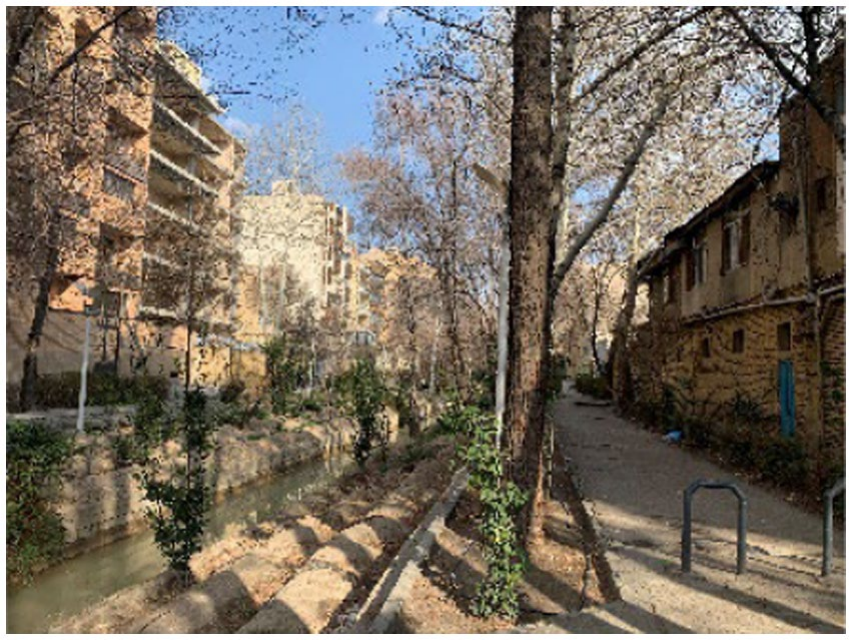
Niasarm Madi (authors).

**Figure 3:**
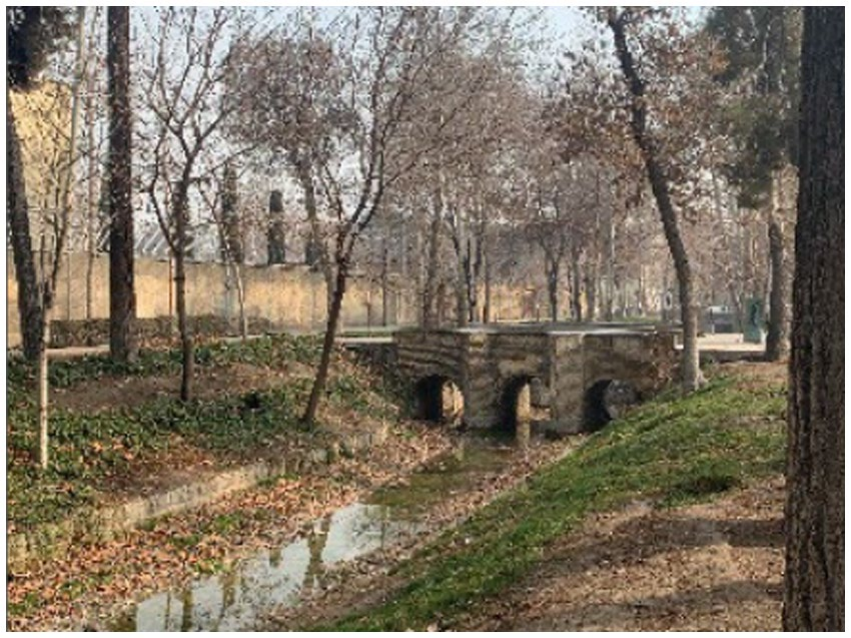
Farshadi Madi (authors).

**Figure 4:**
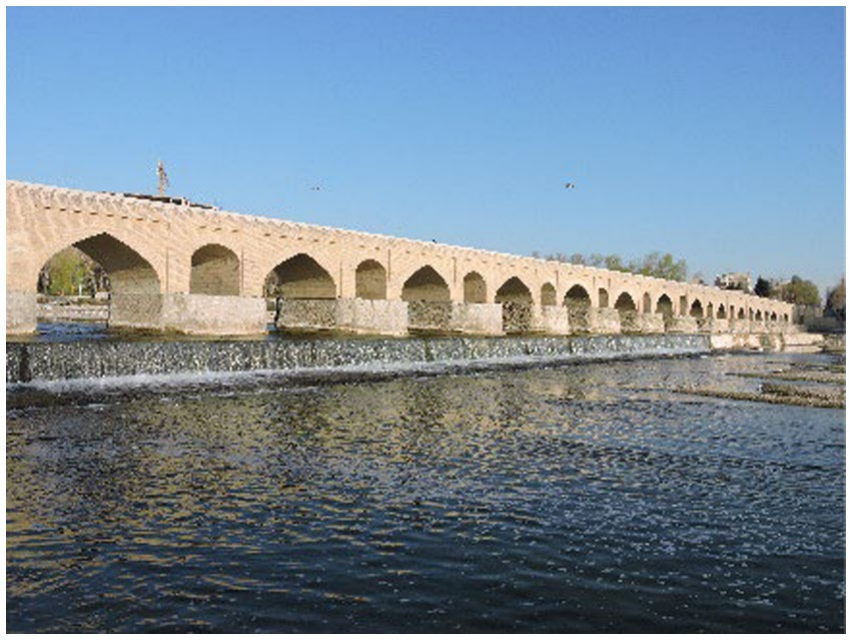
Zayandeh Rood River.

Isfahan, a historic city, is known as the third most populated city in Iran. Zayandeh Rood River and the green public spaces and parks alongside this river are some of the most important landmarks in this city, creating a favorable green and blue space and significantly influencing the citizens’ mental health (Figures 2–4). Transferring the water of this river to gardens, agricultural lands, and neighborhoods through canals called Madi in the local language has developed green corridors in many city districts (59). As stated by the Isfahan municipality in 2021, the UGSs in the city are 28.17 square meters *per capita* (60).

**Figure 5:**
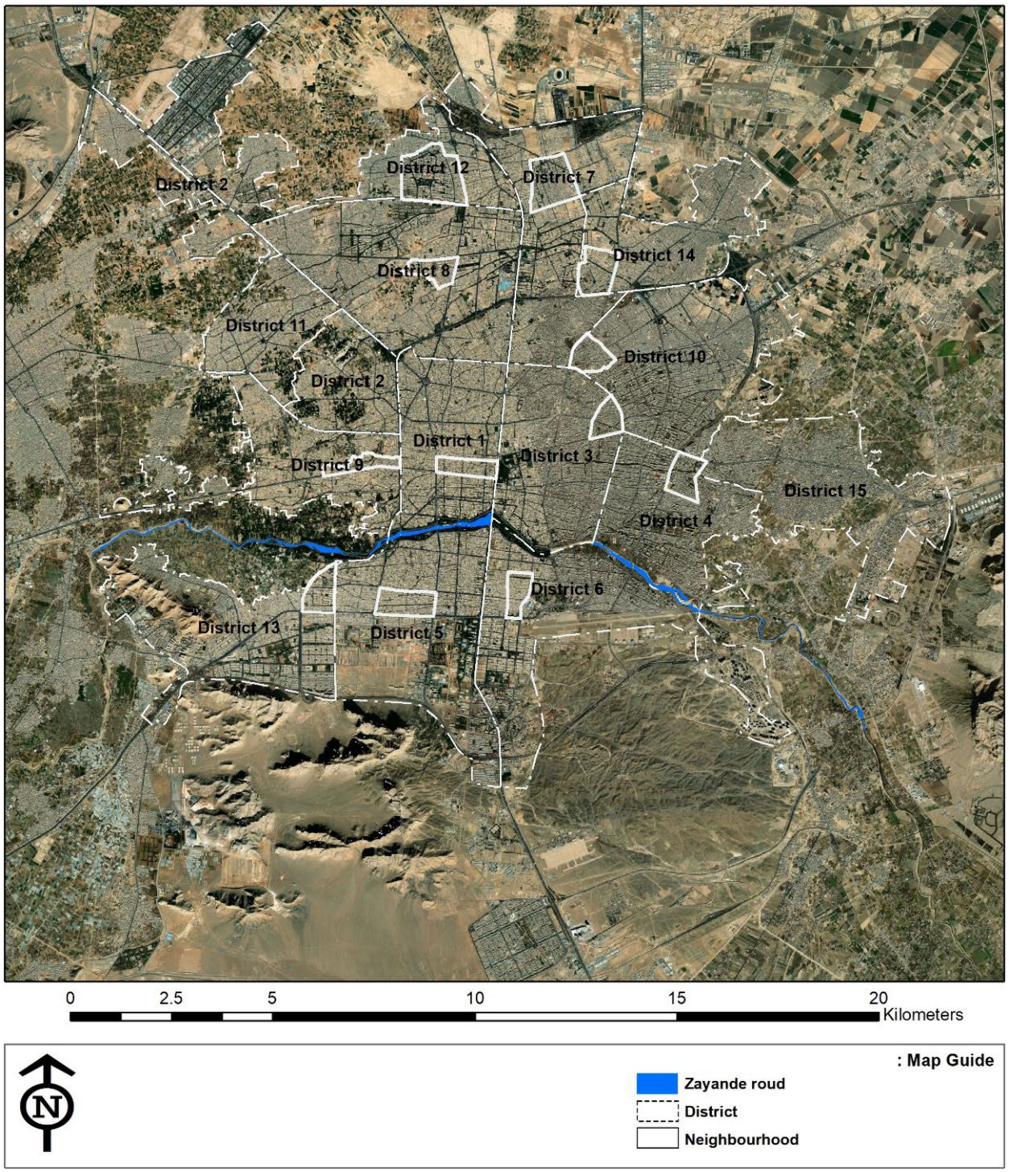
The location of the selected neighborhoods in the study.

The youth aged 15–24 at high school and undergraduate levels were asked to complete a self-reported questionnaire through Google Forms platform. As previous studies have stated, the socio-economic characteristics of the neighborhoods are related to residents’ mental health and their perception of UGS (61–63). In our study, this variable was controlled by selecting neighborhoods with similar socio-economic status. Based on the latest comprehensive plan (2017), Isfahan has 15 municipalities that have been divided into several neighborhoods. Based on the socio-economic data of the comprehensive plan (64), the questionnaire was distributed within 12 neighborhoods with similar socio-economic status across the city to cover the variety of participants. Therefore, the online questionnaire link was distributed to active youth groups within the designated neighborhoods through the social department of the Isfahan city municipality. Completing the online questionnaire proved instrumental, particularly in the post-COVID-19 era with insufficient vaccination coverage, as face-to-face interactions with youth in the neighborhoods were not feasible. By prioritizing the maintenance of social distance and opting for an online approach, we avoided potential biases in the data collection process. Figure 5 shows the location of the selected neighborhoods and Table 1 presents the socio-demographic characteristics of the residents of these neighborhoods.

**Table 1:** Socio-demographic status of the selected neighborhoods [source: (60, 64)].

Neighborhood number on the map	Region	Neighborhood name	Male	Female	Total population	Gender ratio*	Employed residents	Employment to population ratio (%)	Educated residents**	Educated residents to population ratio (%)
1	1	Khalja	2,764	3,034	5,798	91	1,674	29	2,107	36
2	3	Ahmad Abaad	3,191	3,180	6,370	100	1871	29	1,380	21
3	4	Hamedanian	4,520	4,504	9,024	100	2,482	27	1985	22
4	5	Hossein Abaad	6,627	7,352	13,979	90	3,840	27	5,397	39
5	6	Mosalla Masjed	4,123	4,470	8,593	92	2,324	27	2,756	32
6	7	Kaveh town	8,520	8,297	16,817	103	4,708	28	2,898	17
7	8	Razmandegah	4,502	4,486	8,987	100	2,744	30	2,191	24
8	9	Baharanchi	1742	1779	3,521	98	1,080	31	665	19
9	10	Mosalla	4,310	4,301	8,611	100	2,428	28	1,175	14
10	12	Malekshahr	17,367	18,149	35,516	96	10,626	30	9,152	26
11	13	Baghziyar	5,619	5,755	11,374	98	3,309	29	3,258	29
12	14	Shahpasand	6,473	6,206	12,679	104	3,582	28	2,296	18

*The number of males per 100 females.

**Residents with a high school diploma or higher degree.

A total of 273 questionnaires were collected. The Google Forms questionnaire could only be registered when all the items were completed, there was no missing data, and the exact number of incomplete submissions is unknown. The recommended sample size for structural equations, as per Kline (65), is 10–20 times the number of parameters. With 26 parameters in our research model, the acceptable sample limit is 260. Therefore, the 273 completed questionnaires collected align with the criteria, constituting an acceptable sample size for structural equations analysis. The Ethics Board of Iran University of Medical Sciences (IUMS) approved the project. Besides, informed consent was obtained from all subjects.

### 3.2 Instruments and measurements

Our study aims to discover the role of UGSs in the youth’s mental health and their attitude toward UGSs in the post-COVID-19 era. Therefore, a questionnaire containing 26 questions about UGSs and mental health was developed. Questions were designed using a conceptual framework extracted from the desk study. Ten academics and professionals approved this questionnaire’s face and content validity. The Cronbach’s alpha was 0.76, and ICC = 0.913. The final questionnaire consisted of six parts, described below:

#### 3.2.1 Perceived UGSs in residential areas

Utilizing the approach employed in the study by Liu et al. (67), Yang et al. (68), and Li et al. (26), we measured people’s perception of UGS in the neighborhood to assess its correlation with mental health. Therefore, we asked the youth to answer, “How much urban green space (e.g., trees, plants, etc.) is in your neighborhood”?” Answers were given using a 5-point Likert scale, and the participants chose from 1 = very little to 5 = very much based on their experience.

#### 3.2.2 Perceived pollution of UGSs

Based on the previous studies (26), the youth were asked to assess the perceived air and noise pollution in the UGSs based on a 5-point Likert scale: “How do you evaluate the level of noise pollution (e.g., honks, car engines, etc.) in the UGSs in the last month?” and “How do you evaluate the level of air pollution (smoke and dust) in the UGSs in the last month”?” (Cronbach *α* = 0.75). The mean score of the questions was used as the total score of this variable.

Based on the Isfahan municipality, it has been reported that there were about 23 days of healthy air (51–100 AQI) and 8 days of unhealthy air for sensitive groups (101–150 AQI) in September 2022 (66). In summary, air pollution in September was minimal and did not adversely impact individuals’ perceptions while completing the questionnaire. Typically, air pollution in Iran’s metropolises, including Isfahan, tends to escalate toward the end of November and persists through December (67).

#### 3.2.3 Perceived aesthetics of UGSs

To assess individuals’ perception of the aesthetics of spaces, we employed the questions from the adapted version (68) of the NEWS questionnaire (69). These questions measure people’s perceptions of the environment’s aesthetics while walking. We asked the participants to express their perceptions about the attractiveness and aesthetics of UGSs. “Do you consider UGSs near your residence well-designed, clean, well-maintained, and furnished?.” “In this area, are there any historic and attractive buildings with appropriate or considerable architecture?” In this area, are there any attractive landscapes (e.g., natural greenness, the presence of water in any forms, views of the mountains or other natural landscapes”)?” and “How do you rate the beauty of UGSs near your residence”?” The youth answered these questions using the 5-point Likert scale, and the Cronbach *α* was 0.77. We used the mean score of the questions as the total score of this variable.

#### 3.2.4 Fear of COVID-19 infection

Due to the high mortality rates of COVID-19 globally, many people have suffered from the fear of COVID-19 transmission outside their homes. We formulated three questions assessing individuals’ fear of being in open spaces and contracting COVID-19 using The Fear of COVID-19 Scale (FCV-19S) questionnaire (70). Therefore, we asked the following three questions to find the relation between perceiving the UGS of the neighborhood and the fear of COVID-19 infection. “To what extent are you afraid of getting infected with COVID-19 in crowded spaces in UGSs, such as playgrounds, around fountains, bridges, sitting areas, and public toilets?” “When you think about COVID-19, do you feel an increase in your heart rate, insomnia, or any mental disorders?” and “How much does the possibility of reoccurrence of high infection of COVID-19 make you uneasy?” Answers are categorized from 1 = very little to 5 = very much. Cronbach α is calculated at 0.77. We used the mean score of the questions as the total score of this variable.

#### 3.2.5 Feeling anxiety

The Generalized Anxiety Disorder 7-item scale (GAD-7) was used to assess the anxiety level of the participants (74). In seven questions, the youth were asked to state how much they suffered from anxiety symptoms in the past 2 weeks using UGSs. The answers were based on a 4-point Likert scale (0 = not at all to 3 = nearly every day). The total score indicated the respondents’ anxiety level (0–4 = minimal anxiety, 5–9 = mild anxiety, 10–14 moderately severe anxiety, and 15–19 = severe anxiety). There was a high reliability of Cronbach *α* estimated at 0.82.

#### 3.2.6 Feeling depression

The 9-item Patient Health Questionnaire (PHQ-9) was used to assess the level of depression of the participants (71). In these nine questions, the symptoms of depression, lack of pleasure, hopelessness, sleep problems, fatigue, changes in appetite, and thoughts of suicide in the youth were measured, and they were asked to answer the questions based on a 4-point Likert scale (0 = not at all to 4 = almost every day). The scores were considered in five categories: 0–4 = minimal depression, 5–9 = mild depression, 14–10 = moderate depression, 15–19 = moderately severe depression, and 20–27 = severe depression with Cronbach α = 0.85.

### 3.3 Data analysis

In our study, Spearman’s Rank-Order Correlation (72) was done to examine the association between the variables, including perceived UGSs, perceived pollution, perceived aesthetics, fear of COVID-19 infection, and mental disorders (anxiety and depression) as well as age and gender of the participants. Data analysis was done with IBM SPSS Statistics software, version 26. If the correlation between the variables is strong, it is closer to +1 or − 1, and if two variables are independent, this number is closer to zero (73).

The structural equation model was employed to examine the hypothesized pathways in the conceptual framework presented in Figure 1 to analyze the relationship between perceived UGSs and youth mental health. A multivariate analysis method includes several dependent and independent variables in a single model and two categories of latent and observed variables (65). Perceived UGS was considered an independent variable, and others were dependent. Depression and anxiety were considered dependent variables obtained through the standardized questionnaires of GAD-7 and PHQ-9. Perceived pollution is a latent variable with two observed variables: air pollution and noise. Perceived aesthetics and fear of COVID-19 infection are the other observed variables in this model. As illustrated in Figure 1, demographic characteristics, including age and gender, were considered control variables in this model as relative quantitative and nominal qualitative variables, respectively.

## 4 Results

### 4.1 Characteristics of the participants

Two hundred and seventy-three youths between 15 and 24 years old in Isfahan were asked to answer the questionnaire. Table 2 displays that the mean age was 20.22, and about 52% of the participants were female. Also, 15 and 17.6% of the participants reported symptoms of moderate anxiety and moderate depression, respectively.

**Table 2:** Characteristics of the participants (*n* = 273).

Variable	Category	Mean (S.D.)	Percentage (*n*)
Gender	Male		45.8% (125)
	Female		54.2% (148)
Age (year)		20.22 (2.9)	
	15–18 (Highschool student)		30.8% (84)
	19–24 (University student)		69.2% (189)
Perceived UGS	Little		11.4% (31)
	A little		7.3% (20)
	Moderate		12.5% (34)
	Much		32.2% (88)
	Very much		36.6% (100)
Perceived pollution	Little		12.5% (34)
	A little		50.5% (138)
	Moderate		18.3% (50)
	Much		11.7% (32)
	Very much		7.0% (19)
Perceived aesthetic	Little		1.5% (4)
	A little		14.3% (39)
	Moderate		24.2% (66)
	Much		43.2% (118)
	Very much		16.8% (46)
Fear of COVID-19 infection	Little		15.8% (43)
	A little		44% (120)
	Moderate		24.5% (67)
	Much		11.7% (32)
	Very much		4.0% (11)
Anxiety	–	7.43% (3.69)	
	Minimal anxiety		24.5% (67)
	Mild anxiety		53.1% (145)
	Moderate anxiety		15.0% (41)
	Severe anxiety		7.3% (20)
Depression	–	8.02% (4.61)	
	Minimal depression		28.2% (77)
	Mild depression		45.1% (123)
	Moderate depression		17.2% (47)
	Moderately severe depression		7% (19)
	Severe depression		2.6% (7)

### 4.2 Correlation between variables

Table 3 displays that “anxiety” and “depression” were positively correlated with perceived noise, air pollution, and fear of COVID-19 infection. However, these two variables had a negative correlation with perceived UGSs. While there is no significant correlation between anxiety and gender in this statistical population, female youths have reported lower levels of depression than male youths.

**Table 3:** Spearman’s rank correlation coefficient.

Item	1	2	3	4	5	6	7	8
1. Gender (male = 1)	1.0							
2. Age	−0.005	1.0						
3. Perceived fear of COVID-19 infection	−0.147*	0.042	1.0					
4. Perceived UGS	0.11	−0.02	−0.336**	1.0				
5. Perceived noise	−0.066	0.021	0.256**	−0.515**	1.0			
6. Perceived air pollution	−0.056	0.112	0.272**	−0.559**	0.516**	1.0		
7. Anxiety	−0.103	0.043	0.336**	−0.655**	0.555**	0.539**	1.0	
8. Depression	−0.174**	−0.034	0.345**	−0.562**	0.476**	0.526**	0.614**	1.0

*, *p* < 0.05; **, 0.01.

### 4.3 Results of the SEM analysis

Based on the theoretical background and bivariate correlation, pathways between variables were proposed and analyzed in IBM SPSS Amos 24 software. The model fit indices published by West et al. were used to check the goodness of fit. Goodness-of-fit index (GFI) >0.95; standardized root mean square residual (RMSEA)<0.8; comparative fit index [(CFI) >0.95] (74) and discrepancy divided by degree of freedom ((CMIN/DF) ≤3) were considered good indicators (65). In the M0 model, we checked the confirmatory factor analysis model based on model fit indices and removed the pathways whose *p*-value of regression weights was insignificant. Then, in the M1 model, we added demographic characteristics, including age and gender to the model. Finally, we employed the structural equation model in the M2 model using the reduced model to demonstrate the regression paths between the exogenous variable, perceived UGSs, and other endogenous variables, including perceived pollution, perceived aesthetics, fear of COVID-19 infection, anxiety, and depression (CMIN/DF = 1.94, GFI = 0.97, CFI = 0.98, RMSEA = 0.06).

**Figure 6:**
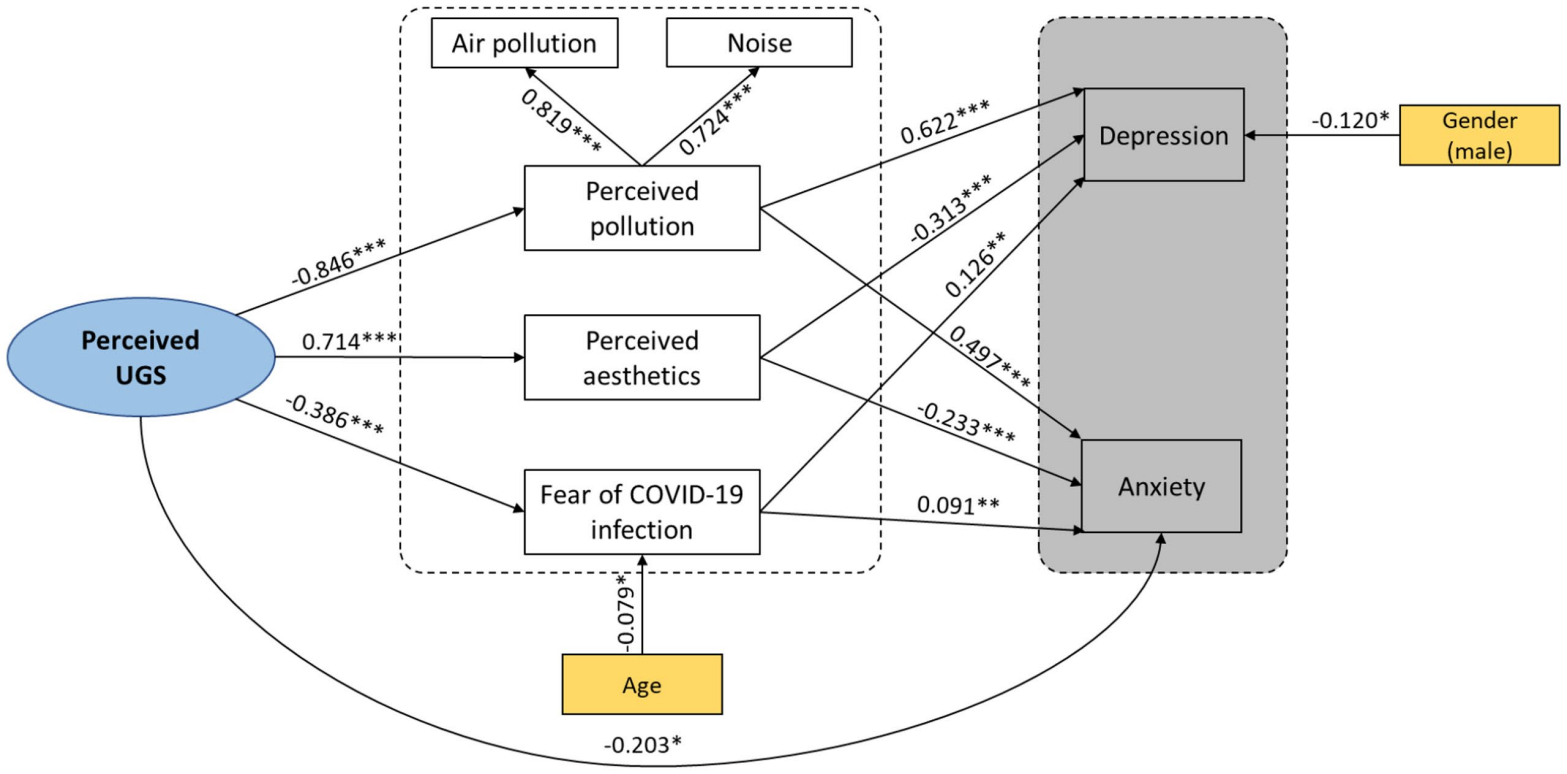
Final structural equation model (M2) with standard regression weight (β). *, *p*< 0.05; **, *p*<0.01; ***, *p* = 0.00.

In the final SEM model, although there was no significant direct pathway between perceived UGSs and depression (*β* = 0.12, *p* = 0.304), all other pathways between the variables were shown to be significant (65). Despite a significant direct pathway between depression and gender (*β* = −0.12, *P* = -0.043) and fear of COVID-19 infection with age (*β* = −0.079, *p* = 0.031), these pathways were relatively deemed weak. Moreover, the correlations between fear of COVID-19 infection and depression (*β* = 0.126) and anxiety (*β* = 0.091) were weak (Figure 6).

Table 4 illustrates that perceived UGSs (as the independent variable) had a significant total effect on other variables in our study, demonstrating the direct and indirect impacts of perceived UGSs on the youth’s mental health. The total effect of perceived UGS on perceived pollution, perceived aesthetics, and fear of COVID-19 infection was utterly from the direct effect. Inversely, the total effect on depression is entirely from the indirect effect. The total effect of perceived UGSs on anxiety mainly resulted from the indirect effect.

**Table 4:** The standardized total, direct, and indirect effects of perceived UGSs on the core variables.

Variable	Total β (95% CI)	*p* value	Direct β (95% CI)	*p* value	Indirect β (95% CI)	*p* value
Perceived pollution	−0.83 (−0.89, −0.77)	0.003	−0.86 (−0.89, −0.77)	0.003	–	–
Perceived aesthetics	0.71 (0.65, 0.77)	0.005	0.71 (0.65, 0.77)	0.005	–	–
Fear of COVID-19 infection	−0.38 (−0.5, −0.26)	0.004	−0.38 (−0.5, −0.26)	0.004	–	–
Anxiety	−0.82 (−0.86, −0.78)	0.004	−0.24 (−0.38, −0.05)	0.14	−0.58 (−0.78, −0.45)	0.003
Depression	−0.69 (−0.74, −0.63)	0.005	–	–	−0.69 (−0.74, −0.63)	0.005

## 5 Discussion

This study examines the relationship between perceived UGSs, potential mediators, and the mental health of the youth (15–24 years old) in Isfahan City in the post-COVID-19 era. The results demonstrate that perceived UGSs are associated with youth anxiety through a direct adverse pathway. Therefore, it can be concluded that the youth exposed to UGSs have experienced better mental health conditions than their peers, emphasizing the role of UGSs in improving the youth’s mental health, as mentioned in previous studies (75–78).

Yet, the pandemic has significantly affected daily habits and behaviors in urban spaces worldwide (3) due to social distancing and quarantine. Thus, there was a global limitation for the youth’s access to UGSs for physical and social activities, directly and indirectly affecting their mental health (20). Although in the post-COVID-19 era, the youth’s access to UGSs seems unchecked, the fear of COVID-19 infection can still be considered a hindrance factor. However, in a longitudinal study, the researchers found that the worries about social distancing and COVID-19 infection decreased in the second and third waves compared to the first one in the U.K. (79). In another study, 46.8% of respondents claimed that fear of infection is the second reason after governmental restrictions prevent them from visiting UGSs (78). Based on the SEM analysis of our study, fear of infectious disease has a significant direct pathway to depression in the youth, although they have been started using UGSs. Similarly, in a study investigating the role of inside and outside greenery in depression in Shanghai, the researchers pointed out that UGSs are directly associated with less fear of COVID-19 infection and fewer symptoms of depression (80) in the post-COVID-19 era.

In our study, some participants reported moderate to severe levels of anxiety and depression, which is in agreement with the research (23) conducted after the pandemic. In a study conducted on 332 students in the city of Plovdiv, Bulgaria, around 34.7% of the youth suffered from depression (PHQ ≥ 10), and 21.7% of them suffered from anxiety (GAD≥10) as well. Meanwhile, in our research, these numbers show 26.8 and 22.3%, respectively. Accordingly, it might be concluded that although COVID-19 infection and mortality have globally decreased significantly, the symptoms of anxiety and depression are still common among the youth.

Based on our findings, perceived pollution is associated with the youth’s mental health. Reducing pollution could help heal mental health disorders (81), while UGSs can reduce air pollution and noise (55) by absorbing air pollutants (82), acting as a buffer (54). Our study confirms that pollution is negatively associated with perceived UGSs and positively associated with anxiety and depression. These findings contribute to understanding the factors establishing a connection between UGSs and mental health. Similarly, other studies have found that pollution mediates between UGSs and mental health (15, 26, 51, 57).

According to some studies (83, 84), environmental qualities help more visits to UGSs, and even during the pandemic, the importance of environmental qualities for youth activities in UGSs has been significant (55). The perceived aesthetic mediator is considered a critical quality for likable UGSs. Our results also show that perceived aesthetics can make a connection between using UGSs and decreasing anxiety. Consistently, previous studies also investigated that this mediator links UGSs to psychological well-being (9). For example, research in 2020 on three Chinese cities shows that improving landscape characteristics, including water, trees, and microclimate, can positively affect mental health (85).

Moreover, in disagreement with another study (26), we could not find any significant direct pathway between perceived UGSs and depression. This variable is related to UGSs through indirect pathways. Additionally, our results show a significant direct pathway between anxiety and UGS quality. These unusual results, as mentioned above, can be justified by the mental health status of the participants, who were not depressed but suffered from fear of being infected in public places (86).

Finally, our results revealed the significant direct association pathways between UGSs and other mediators, including perceived pollution, perceived aesthetics, and fear of infectious disease, emphasizing the role of UGSs on youth mental health. In line with the research conducted by John Newton in Australia, he found anxiety caused by fear of death felt by 22% of the respondents due to COVID-19 (87). Besides, the two mediators of perceived pollution and aesthetics showed a robust association (*β* = 0.714) (65) between perceived UGSs and youth mental health with a higher regression coefficient than the fear of being infected. Therefore, as depicted in Figure 6, it can be concluded that the connection between perceived Urban Green Space (UGS) and mental health is mediated by the perceived aesthetic of UGS. Prior research findings have consistently highlighted the significance of UGS features such as beauty, naturalness, cleanliness, well-maintenance, and attractive landscapes and buildings in influencing people’s mental health (63, 88, 89).

A limitation in arid regions is the less availability of UGSs. Isfahan, situated on the periphery of Iran’s central desert and characterized by an arid climate, faces challenges in providing adequate UGSs. This study underscores the significance of UGSs in arid climates, particularly during the COVID-19 pandemic. During this period, with social distancing measures and widespread quarantine, neighborhood UGSs emerged as vital for enhancing mental health and averting the exacerbation of conditions like depression and anxiety among residents. This consideration, supported by prior studies in similar climates, underlines the significance of UGS in future research within such regions.

Recent research in Saudi cities with arid climates indicates that, despite a decline in park visitors during the COVID-19 pandemic, urban parks remain crucial for people’s mental health and well-being. Emphasizing the importance of these parks’ quality and quantity is underscored (93). Kim and Coseo’s study in Phoenix, Arizona, underscores the vital role of urban parks as green infrastructure in mitigating air pollution and promoting public health (90). A study in Greece, characterized by a predominantly dry climate, examines public perception regarding the importance of urban green space (91). Akbar et al.’s research in Yazd, an arid region in Iran, explores the influence of large green spaces on public perception, highlighting the absence of green space as a significant barrier to Urban Green Spaces (UGS). The study also addresses the impact of UGS proximity, the presence of water, and green elements on changing people’s moods (96). In Birjand, a study amid the COVID-19 pandemic revealed a shift in green space usage. Public park visits decreased due to government restrictions, but private green spaces like yards and gardens saw increased activity. The findings underscore the importance of enhancing the quality and quantity of green space in arid cities, emphasizing positive impacts on the relationship with nature, mental health, and recreational opportunities during a pandemic (92).

However, there are still some limitations in our study. The first limitation is the restricted understanding of resident’s interaction with UGSs. Unlike many other studies exploring the correlation between UGS interaction and mental health (93), this research focuses on resident’s perceptions of UGSs in neighborhoods.

Bias is considered another limitation of our study in data collection with a self-administrated questionnaire because of the problem of underestimating the questions among participants. Yet, at the beginning of the questionnaire, we described the study for the respondents and categorized anxiety and depression questions with a specific title the youth may underreport due to the stigma, fear, and possibility of labeling that such topics have among the people (3). Also, this research is cross-sectional, and we could not investigate the role of time in our target group. Finally, the average statistical population may not be generalizable to all youth. The SEM model may also lack sufficient power to detect other potentially essential pathways.

Certainly, subjective research is not without its limitations, Numerous studies emphasize the importance of investigating research related to the environment, its connection with humans, and its impact on health from both objective and subjective perspectives (94–96). This dual approach is advocated to enhance the depth of research in this domain. Therefore, a comprehensive exploration of this field requires an examination of not only the subjective dimension but also the objective one. By incorporating both perspectives, researchers can provide a more holistic understanding of the complexities inherent in the relationship between the environment and human health.

## 6 Conclusion

This study investigates the relationship between perceived UGSs and youth mental health in Isfahan based on a conceptual framework demonstrating the relationship between different variables through the mediators of perceived pollution, perceived beauty, and fear of COVID-19 infection in the post-COVID-19 era. The youth with more opportunities to be exposed to more UGSs generally had better mental health and fewer symptoms of depression and anxiety. The structural equation model shows the potential pathways between UGSs and mental health among the youth. Less pollution, better environmental conditions, and less fear of COVID-19 infection can help decrease anxiety and depression symptoms. Since this research was cross-sectional, future studies can be longitudinal to determine whether the UGSs can reduce the symptoms of anxiety and depression caused by COVID-19 over time. Due to the nature of our cross-sectional data, a causal relationship between the analyzed variables could not be proven; nonetheless, our model suggests that improved UGS quality, such as less noise and air pollution, can boost mental health. Future urban public design projects should improve environmental qualities to safeguard urban residential spaces and opportunities for UGSs for physical and recreational activities for all citizens.

## Data availability statement

The raw data supporting the conclusions of this article will be made available by the authors, without undue reservation.

## Ethics statement

The studies involving humans were approved by the Shahid Beheshti University of Medical Sciences. The studies were conducted in accordance with the local legislation and institutional requirements. The participants provided their written informed consent to participate in this study. Written informed consent was obtained from the individual(s) for the publication of any potentially identifiable images or data included in this article.

## Author contributions

MM: Data curation, Formal analysis, Resources, Software, Writing – original draft. PH: Conceptualization, Investigation, Methodology, Supervision, Visualization, Writing – review & editing. AL: Methodology, Resources, Supervision, Writing – review & editing.
