# Supplementary material for: Perceived urban green spaces and youth mental health in the post-COVID-19 era
Source: Front Public Health. 2024 Feb 7;12:1265682. doi: 10.3389/fpubh.2024.1265682 (PMC10879616; doi:10.3389/fpubh.2024.1265682)
Supplement: Supplementary file 1 [file Data_Sheet_1.PDF]

This questionnaire is designed to investigate the role of green space in the mental health of young people in Isfahan.

**Gender:**	○ Female	○ Male
**Age range:**	○ 15-18	○ 19-24

**Part 1**	Very little	Below the average	Average	Above average	Very much
1.How much urban green space (e.g., trees, plants, etc.) is in your neighborhood?	○	○	○	○	○
2.How do you evaluate the level of noise pollution (e.g., honks, car engines, etc.) in the UGSs in the last month?	○	○	○	○	○
3.How do you evaluate the level of air pollution (smoke and dust) in the UGSs in the last month?	○	○	○	○	○
4.Do you consider UGSs near your residence well-designed, clean, well-maintained, and furnished?	○	○	○	○	○
5.In this area, are there any historic and attractive buildings with appropriate, or considerable architecture?	○	○	○	○	○
6.In this area, are there any attractive landscapes (e.g., natural greenness, the presence of water in any form, views of the mountains or other natural landscapes)?	○	○	○	○	○
7.How do you rate the beauty of UGSs near your residence?	○	○	○	○	○
8.To what extent are you afraid of getting infected with COVID-19 in crowded spaces in UGSs, such as playgrounds, around fountains, bridges, sitting areas, and public toilets?	○	○	○	○	○
9.When you think about COVID-19, do you feel an increase in your heart rate, insomnia, or any mental disorders?	○	○	○	○	○
10.How much does the possibility of reoccurrence of high infection of COVID-19 make you uneasy?	○	○	○	○	○

**Part 2:** Over the last two weeks, how often you've been bothered by the following problems?<br>**Generalized Anxiety Disorder (GAD-7)**	Not at all	Several days	More than half the days	Nearly every day
1.Feeling nervous, anxious, or on edge	○	○	○	○
2.Not being able to stop or control worrying	○	○	○	○
3.Worrying too much about different things	○	○	○	○
4.Trouble relaxing	○	○	○	○
5.Being so restless that it is hard to sit still	○	○	○	○
6.Becoming easily annoyed or irritable	○	○	○	○
7.Feeling afraid as if something awful might happen	○	○	○	○
**Patient Health Questionnaire (PHQ-9)**	○	○	○	○
1.Little interest or pleasure in doing things	○	○	○	○
2.Feeling down, depressed, or hopeless	○	○	○	○
3.Trouble falling or staying asleep, or sleeping too much				
4.Feeling tired or having little energy	○	○	○	○
5.Poor appetite or overeating	○	○	○	○

	6.Feeling bad about yourself – or that you are a failure or have let yourself or your family down				
	7.Trouble concentrating on things, such as reading the newspaper or watching television				
	8.Moving or speaking so slowly that other people could have noticed? Or the opposite – being so fidgety or restless that you have been moving around a lot more than usual				
	9.Thoughts that you would be better off dead or of hurting yourself in some way				
